# Cost-effectiveness of differentiated care models that incorporate economic strengthening for HIV antiretroviral therapy adherence: a systematic review

**DOI:** 10.1186/s12962-024-00557-w

**Published:** 2024-05-24

**Authors:** Annie Liang, Marta Wilson-Barthes, Omar Galárraga

**Affiliations:** 1 Brown University School of Public Health, Providence, RI USA; 2 Department of Epidemiology, Brown University School of Public Health, Providence, RI USA; 3 Department of Health Services, Policy and Practice; and International Health Institute, Brown University School of Public Health, 121 South Main Street, Box G-S121-2, Providence, RI USA

**Keywords:** Differentiated care, Differentiated service delivery, Economic strengthening, Microfinance, Conditional cash transfer, Cost-effectiveness, HIV, Antiretroviral therapy, Sub-Saharan Africa

## Abstract

**Background:**

There is some evidence that differentiated service delivery (DSD) models, which use a client-centered approach to simplify and increase access to care, improve clinical outcomes among people living with HIV (PLHIV) in high HIV prevalence countries. Integrating economic strengthening tools (e.g., microcredit, cash transfers, food assistance) within DSD models can help address the poverty-related barriers to HIV antiretroviral therapy (ART). Yet there is minimal evidence of the cost-effectiveness of these types of multilevel care delivery models, which potentially prohibits their wider implementation.

**Methods:**

Using a qualitative systematic review, this article synthesizes the literature surrounding the cost-effectiveness of differentiated service delivery models that employ economic strengthening initiatives to improve HIV treatment adherence in low- and middle-income countries. We searched three academic databases for randomized controlled trials and observational studies published from January 2000 through March 2024 in Sub-Saharan Africa. The quality of each study was scored using a validated appraisal system.

**Results:**

Eighty-nine full texts were reviewed and 3 met all eligibility criteria. Two of the three included articles were specific to adolescents living with HIV. Economic strengthening opportunities varied by care model, and included developmental savings accounts, microenterprise workshops, and cash and non-cash conditional incentives. The main drivers of programmatic and per-patient costs were ART medications, CD4 cell count testing, and economic strengthening activities.

**Conclusion:**

All economic evaluations in this review found that including economic strengthening as part of comprehensive differentiated service delivery was cost-effective at a willingness to pay threshold of at least 2 times the national per capita gross domestic product. Two of the three studies in this review focused on adolescents, suggesting that these types of care models may be especially cost-effective for youth entering adulthood. All studies were from the provider perspective, indicating that additional evidence is needed to inform the potential cost-savings of DSD and economic strengthening interventions to patients and society. Randomized trials testing the effectiveness of DSD models that integrate economic strengthening should place greater emphasis on costing these types of programs to inform the potential for bringing these types of multilevel interventions to scale.

**Supplementary Information:**

The online version contains supplementary material available at 10.1186/s12962-024-00557-w.

## Introduction

Responding to the World Health Organization’s Treat All Policy, low- and middle-income countries (LMICs) are increasingly using differentiated service delivery (DSD) models as a way to rapidly scale up access to life-saving antiretroviral therapy for people living with HIV (PLHIV) [1]. According to the International AIDS Society, “differentiated service delivery (DSD), previously referred to as differentiated care, is a client-centred approach that simplifies and adapts HIV services across the cascade to reflect the preferences, expectations and needs of people living with and affected by HIV, while reducing unnecessary burdens on the health system” [2]. DSD models aim to make care “patient-centered” while reducing logistical and administrative burden(s) on traditional, resource-constrained care facilities [1]. These models have shown to be effective for increasing treatment adherence, but most do not address the persistent poverty-related barriers to HIV care engagement (e.g., long and costly distances to facilities, food insecurity, HIV stigma). A recent systematic review from 20 LMICs found that economic strengthening interventions such as conditional cash transfers, microcredit, and transportation assistance can improve medication adherence and care-seeking behaviors among persons living with HIV, with more moderate impacts on clinical outcomes [3]. Two other systematic reviews found that, on their own, differentiated HIV service delivery approaches in Sub-Saharan Africa (SSA) generally cost the same as or less than standard HIV care in terms of the cost per patient per year from a patient perspective [1, 4]. For providers and health systems, the available economic evidence suggests that DSD models in SSA are not cost saving compared to more traditional facility-based care models [4]. A 2017 modeling study found that differentiated service delivery models aiming to increase access to ART in SSA could yield up to a 17.5% reduction in health system costs and health workforce requirements over 5 years [5]. It remains to be seen whether differentiated service delivery models that additionally aim to address poverty-related barriers to care (e.g., food insecurity, long and costing distances to facilities, restricted access to income-generating opportunities) are cost-effective for patients, providers, or society as a whole [6, 7].

The purpose of this systematic review is to (i) summarize the current evidence surrounding the cost and cost-effectiveness of differentiated HIV service delivery models that include economic strengthening compared to differentiated service delivery without economic strengthening and to standard HIV care, and (ii) offer a conceptual framework that can help future researchers understand the key components influencing the incremental cost-effectiveness of these holistic models for patients and providers.

## Methods

### Eligibility criteria

Our review focused on studies of the cost-effectiveness of differentiated HIV care models that incorporated at least one economic strengthening component. Articles were excluded if they were not a randomized controlled trial or observational study, did not include both an economic strengthening and a differentiated care component for promoting ART adherence, or did not report a standard metric for assessing cost-effectiveness of an ART adherence intervention. Economic strengthening included any activity that aimed to generate individual- or household-level income or wealth, such as microfinance groups, social protection programs, savings accounts, or training in financial literacy or entrepreneurship. Articles that were not peer reviewed, published in English, or conducted in SSA were also excluded. There were no restrictions on the study population in terms of age, gender, or SSA region. During the abstract round of screening if the study fit all other criteria (differentiated service delivery in Sub-Saharan Africa with economic strengthening) but did not mention whether a cost-analysis was performed, the study was included for full text screening to account for ancillary costeffectiveness analyses.

### Information sources & search strategy

We conducted a literature search of articles in PubMed (National Center for Biotechnology Information, Bethesda, Maryland) and EconLit (American Economic Association, Nashville, Tennessee), supplemented by an Internet search of Google Scholar. Prior reviews indicate that DSD interventions have been implemented since the 2000s. Thus, we searched articles published from January 1, 2000 through March 31, 2024 using the terms “HIV or AIDS”, “ antiretroviral therapy”, “economic strengthening”, “differentiated service delivery”, “Sub-Saharan Africa” “cost analysis”, “cost-effectiveness” and “cost-savings”. Literature searched in PubMed used MeSH (Medical Subject Headings) controlled vocabulary to select key search terms. The full search strategy implemented for each database is provided in Additional File 1.

### Selection process

Initial search results were reviewed by one reviewer (AL). Abstracts and main texts of articles that met all eligibility criteria were double reviewed (AL and MWB), with a third reviewer consulted when necessary (OG).

### Data collection process

A data extraction tool was developed to capture the following indicators: study context (e.g., country and region of study), design, population, DSD component(s), economic strengthening activity, costing perspective, main drivers of intervention and per-patient costs, cost-effectiveness metric (e.g., incremental cost-effectiveness ratio), willingness-to-pay threshold (WTP), and a binary indicator of whether the intervention showed to be cost-effective (yes/no). Due to significant heterogeneity across studies in terms of effectiveness and cost-effectiveness outcomes, a meta-analysis was not performed. Search findings were reported following the Preferred Reporting Items for Systematic Reviews and Meta-analyses (PRISMA) guidelines [8].

### Quality assessment

Full texts that were standard health economic evaluations were assessed using the validated Quality of Health Economic Studies (QHES) appraisal system developed by Chiou [9, 10]. The quality of each full text article was assessed based on the sixteen weighted criteria listed in Additional File 2. Weighted scores for each criterion were summed to generate an overall quality score ranging from 0 (extremely poor quality) to 100 (excellent quality). Four quality categories (0–25, 25.1–50, 50.1–75, and 75.1–100) were used with scores > 75 indicating high quality studies [10]. Systematic reviews, micro-costing studies, and qualitative analyses were not scored given our focus on randomized controlled trials (RCTs) and observational studies.

### Conceptual framework

Drawing on the papers included in the review, we adapted an existing conceptual framework to synthesize the key components that could be understood to drive the incremental cost-effectiveness of HIV differentiated service delivery models for SSA health systems.

## Results

### Identified articles

**Fig. 1:**
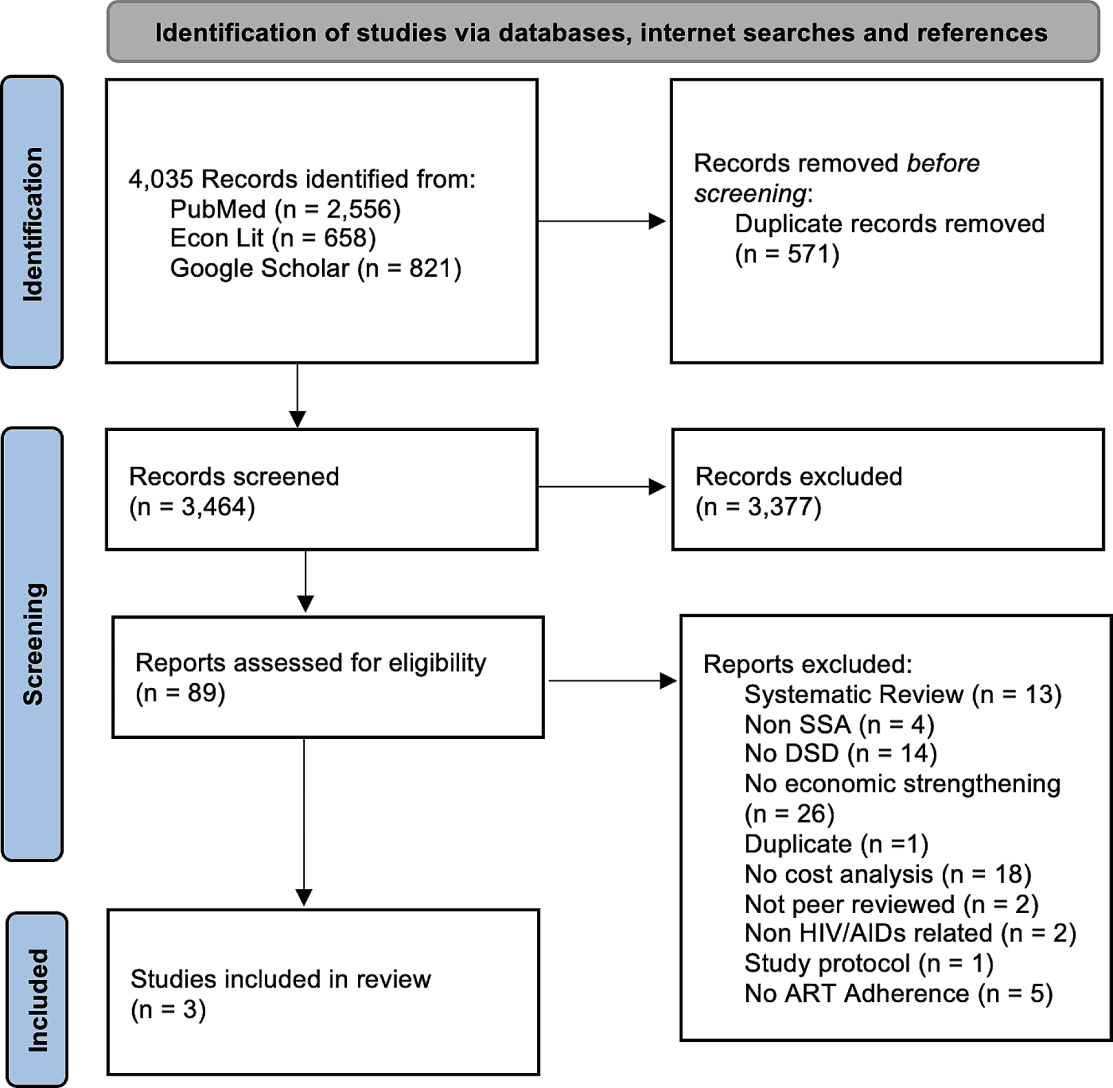
Preferred Reporting Items for Systematic Reviews and Meta-analyses (PRISMA) flow diagram

Figure 1 documents the flow of articles through the review and reasons for exclusion. Most of the 89 articles were peer-reviewed journal articles (93.2%), followed by preprints (2.2%), and scientific reports (2.2%). Of the 57 articles that included a DSD intervention, the most common differentiated service delivery model was community-based ART support and adherence counseling. Of the 40 articles that included an economic strengthening (ES) component, conditional economic (cash and non-cash) incentives and microfinance engagement were the most common ES activities. The most common reasons for exclusion were no economic strengthening component and no cost-effectiveness analysis. Eleven of the 89 reviewed articles were traditional cost-effectiveness analyses and thus were appraised for quality using the Chiou grading system; those that were not appraised using the grading system included costing, budget impact, or other types of non-cost-effectiveness evaluations. The 11 articles had an average quality score of 80.73 (out of 100), and all satisfied at least 11 of the 16 grading criteria (Additional File 2). Of the 89 full text articles that were assessed, three papers met all eligibility criteria and were included in this narrative review.

### Background and summary of included articles

All 3 studies scored above a 75 (out of 100) on the QHES appraisal system, indicating high quality studies [10]. Tozan et al. and Ekwunife et al. scored an 85 on the QHES, satisfying the same criteria. Stevens et al. scored 100 on the QHES, satisfying all criteria. Only Stevens et al. displayed a clear economic model, study methods and analysis, and components of numerator and denominator and justified choice of economic model, main assumptions, and limitations of the study. Although all three included studies were of high quality according to the QHES, each provided minimal rationale for their use of a given economic model which may hinder replicability.

Details of the three included studies are summarized in Table 1. In brief, Tozan et al. [11] estimated the incremental costs of providing additional counseling sessions for HIV and ART adherence as well as an incentivized savings account and workshops on asset building to adolescents living with HIV in Uganda. Incremental intervention costs were compared to the cost of providing routine HIV care and social support alone. Ekwunife et al. [12] estimated the cost-effectiveness of a differentiated care model for young adults living with HIV in Nigeria that included motivational interview sessions and economic incentives based on viral load over 12 months. Stevens et al. [13] modelled the cost-effectiveness of scaling-up a combination care package in Swaziland, which included SMS reminders for ART adherence, counseling and health commodities for ART adherence (e.g., pillboxes and informational materials), and non-cash financial incentives for adults who newly tested positive for HIV. All included studies utilized a facility-based DSD model. For each study, the additional cost for a given intervention compared to the status quo was $970 [95% CI: $508 − 10,275] per additional patient virally suppressed [11], $1,419 per additional patient with undetected viral load [12], and $3,560 per additional quality-adjusted life year (QALY) gained [13].

**Table 1:** Summary of articles included in review: background and effectiveness evidence

Country and source	Study design	Population enrolled	Differentiated Service Delivery model	Economic strengthening activity	Effectiveness outcome
**Uganda (Tozan et al., 2021)**	Two-arm cluster randomized controlled trial (NCT#01790373) at 39 health centers in 5 districts	*N* = 702 adolescents living with HIV (mean age 12 years at enrolment; 56% female) randomized to savings-led family-based economic empowerment ART adherence intervention (*n* = 358) or to bolstered standard of care (*n* = 344).	All trial participants received: - medical care, - psychosocial support on ART resistance and adherence from trained counsellors and peer navigators, and - Uganda-MOH produced communication materials for families. Intervention participants additionally received: − 6 additional counselling sessions on HIV and ART adherence, and - mentorship from peers/research assistants.	Intervention participants received: − 4 workshops on asset building, IGAs and financial saving and planning from non-governmental partner, and - an incentivized child development savings account to which the study provided an initial deposit and matched the adolescent’s monthly savings at a ratio of 1:1 for 24 months.	Proportion of virally suppressed ALWHIV at 24-months (HIV RNA viral load of < 40 copies/mL).
**Nigeria (Ekwunife et al., 2021)**	Two-arm cluster randomized controlled trial (PACTR201806003040425) in 12 health centers	*N* = 246 adolescents living with HIV randomized to receive either conditional economic incentives (CEIs) and motivational interviewing (6 clusters with *n* = 119 patients) or standard HIV care (6 clusters with *n* = 127 patients)	All trial participants received routine HIV care as obtainable in the HIV treatment hospitals. Intervention participants additionally attended motivational interviews (MI) with an adherence counselor trained in MI techniques.	Intervention group participants received US$5.6 when viral load was < 20 copies/ml for the first 3 months, US$2.8 when viral load remained suppressed for the next 3 and 6 months, and US$5.6 if viral load remained < 20 copies/ml for the next 1 year. CEIs were also contingent upon attending motivational interview sessions at each clinic visit.	Difference in proportion of participants with undetected viral load between the two arms (< 20 copies/ml) at month 12 (primary); adherence to ART and hospital appointments, CD4 + count and retention in care (secondary)
**Swaziland (Stevens et al., 2018)**	Two-arm cluster randomized controlled trial (NCT#01904994) in 10 HIV clinics	*N* = 2,197 adults aged ≥ 18 years, newly tested HIV positive (median age 31 years at enrollment, 59% women) received either the combination intervention strategy (5 clusters of *n* = 1,096 participants) or standard HIV care (5 clusters of *n* = 1,101 participants)	All trial participants received routine HIV care. - Intervention participants additionally received: - Point of care CD4 + count testing, - Accelerated ART initiation, - Cellular phone appointment reminders, and - Health education packages.	Non-cash financial incentives in the form of mobile airtime for those linked to care within 1 month of positive test and for completion of 6- and 12-month visits	Linkage to care within 1 month plus retention in care at 12 months after HIV-positive testing (primary); mean time to linkage, assessment for ART eligibility, ART initiation and time to ART initiation, viral suppression (HIV-1 RNA < 1,000 copies/mL at 12 months after HIV testing among patients on ART ≥ 6 months), and loss to follow-up and death at 12 months after HIV testing (secondary)

ALWHIV: Adolescents living with HIV; ART: antiretroviral therapy; IGA: income-generating activity; MOH: Ministry of Health; MI: motivational interviewing

### Cost-effectiveness of differentiated care with economic strengthening

Table 2 presents the cost-effectiveness outcomes from each included study. All analyses used a provider perspective.

**Table 2:** Summary of articles included in review: cost-effectiveness evidence

Country and source	Costing perspective	Top three highest drivers of program cost	Additional cost per patient for intervention delivery	Costs-effectiveness outcome(s)	Cost-effective threshold	National GDP per capita in study year	Was DSD + ES intervention cost-effective?
**Uganda (Tozan et al., 2021)**	Healthcare provider	Child savings account; Mentors; Microenterprise workshops	US$43/year	$970 [95% CI: $508 − 10,275] per additional patient virally suppressed	Not reported	$847.30	Not reported – not cost-effective based on WHO CHOICE threshold of 1x GDP per capita; cost-effective based on threshold of 2–3x GDP per capita
**Nigeria (Ekwunife et al., 2021)**	Healthcare provider	ART medication; Viral load test; CD4 count test	US$166.02/year	$1,419 per additional patient with undetected viral load	US$1,137/QALY gained	$2,027.80	No – based on authors’ WTP threshold Yes – based on WHO CHOICE threshold of 1 to 3x GDP per capita
**Swaziland (Stevens et al., 2018)**	Healthcare provider	ART treatment costs; outpatient care (including incentives); CD4 + count testing	US$60.40/year	$3,560 per QALY gained	US$9,840/QALY gained (3x Swaziland’s GDP per capita)	$3,280.00	Yes

ART: antiretroviral therapy; DSD: differentiated service-delivery; ES: economic strengthening; GDP: gross domestic product; ICER: incremental cost-effectiveness ratio; QALY: quality-adjusted life year; WHO: World Health Organization; WTP: Willingness to pay

The threshold at which a given intervention was deemed cost-effective varied across studies. Tozan et al. did not report a pre-specified willingness to pay threshold [11]. Ekwunife et al. specified a willingness to pay threshold of $1,137 per additional QALY gained by the intervention [12]. Stevens et al. reported a threshold of $9,840 per additional QALY gained (3x Swaziland’s GDP per capita); the Link4Health combination package yielded an incremental cost effectiveness ratio (ICER) of $3,560 per additional QALY gained from the health sector perspective, which the authors deemed cost-effective at a willingness to pay threshold of 3 x Swaziland’s per capita GDP in 2018 [13]. The cost-effectiveness analysis by Ekwunife et al. [12] found that combing conditional economic incentives and motivational interviewing was not cost-effective compared to standard care at the authors’ pre-defined willingness to pay threshold of 0.51 times Nigeria’s per capita GDP; the intervention was cost-effective at 1 x Nigeria’s per capita GDP in 2021 ($2,027.80). Tozan et al. [11] did not report the cost-effectiveness of the combined adherence mentoring and incentivized financial savings account intervention in relation to a pre-defined cost-effectiveness threshold; however the intervention cost less than 2 x Uganda’s per capita GDP ($847.30 in 2021). The respective interventions analyzed by Ekwunife et al. [12] and Tozan et al. [11] were cost-effective (compared to standard care) assuming the World Health Organization’s willingness to pay thresholds of 2 to 3 times the national per capita GDP in the trial year. Across the three studies, the main drivers of programmatic and per-patient costs were ART treatment costs, CD4 cell count testing, and economic strengthening activities including the costs to provide non-financial incentives. In the Uganda cluster-randomized trial [12], the largest cost drivers for the intervention came from viral load tests, CD4 count testing, and patient transportation. Financial incentives and point of care CD4 testing were the main drivers of the observed cost differences in the analysis of the Link4Health cluster-RCT [13]. For Tozan et al. [11], intervention activities including health education sessions, microenterprise workshops, and savings accounts contributed the largest difference in costs between intervention and standard care. All interventions were more expensive than standard care in terms of total cost per patient.

## Synthesizing framework

**Fig. 2:**
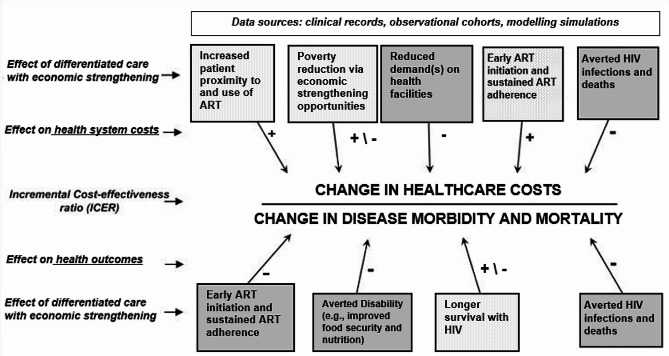
Conceptual Framework. The conceptual framework was adapted from an existing conceptual model developed by Kahn et al. [11] The framework illustrates the key components that can be hypothesized to influence the cost-effectiveness of differentiated HIV care approaches that incorporate economic strengthening activities, from a health system perspective

Based on the three papers in this review, we adapted an existing conceptual model originally developed by Kahn and colleagues [14] to illustrate – from a health system perspective – the key components that can be hypothesized to influence the cost-effectiveness of differentiated service delivery models that incorporate economic strengthening. (Fig. 2) Increasing patient access to antiretroviral therapy immediately following diagnosis and sustaining access over time (e.g., by offering community- or home-based care visits; accelerating ART initiation following point of care CD4 cell count testing) can be expected to add costs to the health system via an increased demand for higher drug quantities, follow-up tests, and personnel time. Similarly, providing economic strengthening opportunities that address known poverty-related barriers to ART adherence will almost always increase the incremental costs of these care delivery approaches if the initiatives are not self-sustaining. For example, providing economic incentives conditional on achieving a viral load below an assay’s lower detection limit will incur additional costs to health ministries who wish to offer this incentive scheme as part of a government social protection program. However, economic strengthening interventions have the potential to be cost-neutral to health systems if they can generate economic growth on their own, as in the case of saving and lending microfinance groups [15, 16] or no fee savings accounts [11]. Averting new HIV infections and decreasing HIV-related morbidity by achieving an undetectable viral load via ART leads to substantial reductions in both disability-adjusted life years and treatment costs. However, as individuals live longer due to ART, they may develop other chronic diseases that incur additional costs to themselves and the health system [17]. Thus, differentiated service delivery models that integrate economic strengthening *and* treatment for co-occurring conditions have the potential to further reduce disease burden without substantially increasing treatment costs.

All elements of this synthesizing conceptual framework are drawn from the authors’ analyses of the supporting literature. Further research on the cost-effectiveness impact of these mechanisms is required to support their validity.

## Discussion

This systematic narrative review found one of three studies testing a differentiated service delivery model that includes economic strengthening to be cost-effective for providers at the authors’ pre-determined WTP threshold. All three included articles were cost-effective at the WHO willingness to pay threshold of at least 2 times a given country’s per capita GDP. Sensitivity analyses [11, 12] and modeling projections [13] in these papers suggest that the cost-effectiveness of these types of multilevel interventions would increase as these care models are brought to scale. Ekwunife et al. [12] found that if CD4 + count tests were performed triannually rather than four times a year, the intervention would become cost-effective. Thus, only minimal adjustments to the differentiated service delivery and ES components could increase the interventions’ cost-effectiveness.

Two of three studies in this review were among adolescents living with HIV. This suggests that cultivating routine medication taking behaviors and establishing positive economic skills (e.g., having a savings account, managing microcredit) may be especially important for lower income adolescents living with HIV who can carry these practices into adulthood. Additionally, two recent feasibility studies did not meet inclusion criteria (i.e., being an RCT or observational study) but were initially screened in this review. Findings from these studies further support the potential of integrating DSD with economic strengthening for improving HIV treatment outcomes along the care continuum (testing, linkage to care, and ART adherence) [18, 19].

The World Health Organization’s Treat-All guidance recommends CD4 testing before initiating antiretroviral therapy (ART) and recommends routine viral load monitoring (over CD4 cell count monitoring) for patients on ART [20, 21]. Viral load monitoring remains the gold standard for monitoring ART adherence and viral suppression among persons living with diagnosed HIV, even in settings where health systems face financial and resource constraints [22–24]. Thus, given that the focus of our review is on cost-effectiveness of models for ART adherence among persons with diagnosed HIV, our findings can inform scale-up of DSD models that support the most widely used HIV treatment outcomes.

Recent protocol studies reveal that there remains space in the literature to continue to examine DSD with economic strengthening interventions as an effective and cost-effective method of enhancing ART adherence [25]. For future research and policymaking, these findings suggest there may be potential for implementing scaled-up DSD with economic strengthening interventions enhancing ART adherence among adolescents and young adults specifically.

Limitations of this systematic review stemmed from the large variability in population, context, and target outcomes across studies, which limited our ability to calculate an overall combined economic effect of these interventions. Additionally, all of the cost-effectiveness analyses in this review calculated cost according to the provider perspective, which limits our ability to quantify the potential economic impact of these combination differentiated care models on patients or society. We aimed to mitigate any potential reviewer bias in the inclusion/exclusion of a quality assessment by using a standardized data extraction tool.

Despite calls for novel cost-effectiveness data of holistic differentiated care models in low- and middle-income countries [1, 6, 26–28], the evidence base surrounding the scale-up potential of DSD interventions and economic strengthening remains sparse. To our knowledge, this is the first review to synthesize the available evidence of poverty-addressing DSD models from a health economics perspective. This evidence is critical for policymakers and health care advocates working to address the economic determinants of HIV treatment adherence with limited resources.

## Conclusion

This brief systematic review demonstrated that including economic strengthening tools as part of differentiated service delivery models is effective and largely cost-effective at common thresholds compared to traditional HIV care. Modelling projections suggest that scaling these types of multilevel intervention may improve their cost-effectiveness in the short and medium term. Future research should consider the cost-effectiveness and cost-savings of these comprehensive HIV care models from a patient and societal perspective.

### Electronic supplementary material

Below is the link to the electronic supplementary material.

Additional file 1: Search syntax

Additional file 2: Quality assessment of full text articles that were standard health economic evaluations

## Data Availability

Data sharing is not applicable to this article as no new datasets were generated or analyzed during the current study.

## Abbreviations

ALWHIV: Adolescents Living with HIV
ART: Antiretroviral Therapy
DSD: Differentiated Service Delivery
ES: Economic Strengthening
GDP: Gross Domestic Product
ICER: Incremental Cost-Effectiveness Ratio
IGA: Income-Generating Activity
LMIC: Low- or Middle-Income Country
MI: Motivational Interviewing
PLHIV: People Living with HIV
PRISMA: Preferred Reporting Items for Systematic Reviews and Meta-Analyses
QALY: Quality-Adjusted Life Year
RCT: Randomized Controlled Trial
SSA: Sub-Saharan Africa
WTP: Willingness-to-Pay

## References

[1] Roy M, Bolton Moore C, Sikazwe I, Holmes CB (2019). A review of Differentiated Service Delivery for HIV Treatment: effectiveness, mechanisms, Targeting, and Scale. Curr HIV/AIDS Rep.

[2] Differentiated Service Delivery. International AIDS Society. 2024. Accessed April 24, 2024. https://www.iasociety.org/ias-programme/differentiated-service-delivery.

[3] Swann M (2018). Economic strengthening for retention in HIV care and adherence to antiretroviral therapy: a review of the evidence. AIDS Care.

[4] Rosen S, Nichols B, Guthrie T, Benade M, Kuchukhidze S, Long L (2022). Do differentiated service delivery models for HIV treatment in sub-saharan Africa save money? Synthesis of evidence from field studies conducted in sub-saharan Africa in 2017–2019. Gates Open Res.

[5] Barker C, Dutta A, Klein K (2017). Can differentiated care models solve the crisis in HIV treatment financing? Analysis of prospects for 38 countries in sub-saharan Africa. J Int AIDS Soc.

[6] Nachega JB, Adetokunboh O, Uthman OA, Knowlton A, Altice FL, Schechter M (2016). Community-based interventions to improve and sustain antiretroviral therapy adherence, Retention in HIV Care and clinical outcomes in low- and Middle-Income Countries for achieving the UNAIDS 90-90-90 targets. Curr HIV/AIDS Rep.

[7] Munyayi FK, van Wyk B, Mayman Y (2022). Interventions to Improve Treatment outcomes among adolescents on antiretroviral therapy with unsuppressed viral loads: a systematic review. IJERPH.

[8] Page MJ, McKenzie JE, Bossuyt PM, Boutron I, Hoffmann TC, Mulrow CD (2021). The PRISMA 2020 statement: an updated guideline for reporting systematic reviews. BMJ.

[9] Chiou CF, Hay JW, Wallace JF, Bloom BS, Neumann PJ, Sullivan SD (2003). Development and validation of a grading system for the quality of cost-effectiveness studies. Med Care.

[10] Spiegel BM, Targownik LE, Kanwal F (2004). The quality of published health economic analyses in digestive diseases: a systematic review and quantitative appraisal. Gastroenterology.

[11] Tozan Y, Capasso A, Sun S, Neilands TB, Damulira C, Namuwonge F (2021). The efficacy and cost-effectiveness of a family-based economic empowerment intervention (suubi + adherence) on suppression of HIV viral loads among adolescents living with HIV: results from a Cluster Randomized Controlled Trial in southern Uganda. JIAS.

[12] Ekwunife OI, Ofomata CJ, Okafor CE, Anetoh MU, Kalu SO, Ele PU (2021). Cost-effectiveness and feasibility of conditional economic incentives and motivational interviewing to improve HIV health outcomes of adolescents living with HIV in Anambra State, Nigeria. BMC Health Serv Res.

[13] Stevens ER, Li L, Nucifora KA, Zhou Q, McNairy ML, Gachuhi A (2018). Cost-effectiveness of a combination strategy to enhance the HIV care continuum in Swaziland: Link4Health. PLoS ONE.

[14] Kahn JG, Marseille EA, Bennett R, Williams BG, Granich R (2011). Cost-effectiveness of antiretroviral therapy for prevention. Curr HIV Res.

[15] Genberg BL, Wachira J, Steingrimsson JA, Pastakia S, Tran DNT, Said JA (2021). Integrated community-based HIV and non-communicable disease care within microfinance groups in Kenya: study protocol for the Harambee Cluster randomised trial. BMJ Open.

[16] Pastakia SD, Manyara SM, Vedanthan R, Kamano JH, Menya D, Andama B (2017). Impact of bridging Income Generation with Group Integrated Care (BIGPIC) on hypertension and diabetes in Rural Western Kenya. J Gen Intern Med.

[17] Negin J, Bärnighausen T, Lundgren JD, Mills EJ (2012). Aging with HIV in Africa: the challenges of living longer. AIDS.

[18] Kim HY, Inghels M, Mathenjwa T (2024). The impact of a conditional financial incentive on linkage to HIV care: findings from the HITS cluster randomized clinical trial in rural South Africa. Preprint medRxiv.

[19] Kibel M, Nyambura M, Embleton L (2023). Enabling adherence to treatment (EAT): a pilot study of a combination intervention to improve HIV treatment outcomes among street-connected individuals in western Kenya. BMC Health Serv Res.

[20] Guideline on when to start antiretroviral therapy and on pre-exposure prophylaxis for HIV. who.int. Published September 1, 2015. Accessed April 24. 2024. https://www.who.int/publications/i/item/9789241509565. 26598776

[21] Brazier E, Tymejczyk O, Zaniewski E (2021). Effects of National Adoption of treat-all guidelines on Pre-antiretroviral Therapy (ART) CD4 testing and viral load monitoring after ART initiation: a regression discontinuity analysis. Clin Infect Dis.

[22] Pham P MD, Nguyen HV, Anderson D, Crowe S, Luchters S. Viral load monitoring for people living with HIV in the era of test and treat: progress made and challenges ahead - a systematic review. BMC Public Health. 2022;22(1):1203. 10.1186/s12889-022-13504-2. Published 2022 Jun 16. 10.1186/s12889-022-13504-2 PMC9202111 35710413

[23] Okoboi S, Musaazi J, King R (2022). Adherence monitoring methods to measure virological failure in people living with HIV on long-term antiretroviral therapy in Uganda. PLOS Glob Public Health.

[24] World Health Organization. Updated recommendations on HIV prevention, infant diagnosis, antiretroviral initiation and monitoring. who.int. Published March 17, 2021. Accessed April 24. 2024. https://www.who.int/publications/i/item/9789240022232. 33822559

[25] van Heerden A, Szpiro A, Ntinga X, Celum C, van Rooyen H, Essack Z, Barnabas R (2023). A sequential multiple assignment Randomized Trial of scalable interventions for ART delivery in South Africa: the SMART ART study. Trials.

[26] Decroo T, Rasschaert F, Telfer B, Remartinez D, Laga M, Ford N (2013). Community-based antiretroviral Therapy Programs can overcome barriers to Retention of Patients and Decongest Health Services in Sub-saharan Africa: a systematic review. Int Health.

[27] Chaiyachati KH, Ogbuoji O, Price M, Suthar AB, Negussie EK, Bärnighausen T (2014). Interventions to improve adherence to antiretroviral therapy: a Rapid systematic review. AIDS.

[28] Creese A, Floyd K, Alban A, Guinness L (2002). Cost-effectiveness of HIV/AIDS interventions in Africa: a systematic review of the evidence. Lancet.

