# Supplementary material for: Cost-effectiveness of differentiated care models that incorporate economic strengthening for HIV antiretroviral therapy adherence: a systematic review
Source: Cost Eff Resour Alloc. 2024 May 24;22:46. doi: 10.1186/s12962-024-00557-w (PMC11127452; doi:10.1186/s12962-024-00557-w)
Supplement: Supplementary file 1 — Additional file 1: Search syntax [file 12962_2024_557_MOESM1_ESM.docx]

Additional File 1 for the manuscript:

**Full Title:** Cost-effectiveness of differentiated care models that incorporate economic strengthening for HIV antiretroviral therapy adherence: a systematic review

**Running Title:** Cost-effectiveness of differentiated HIV care

**Authors:** Annie Liang ^1^; Marta Wilson-Barthes^2^; Omar Galárraga^3,§^

^1^ Brown University School of Public Health, Providence, RI United States; [annie_liang@brown.edu](mailto:annie_liang@brown.edu)

^2^ Department of Epidemiology, Brown University School of Public Health, Providence, RI United States; [marta_wilson-barthes@brown.edu](mailto:marta_wilson-barthes@brown.edu) [ORCID ID: 0000-0002-9845-7142]

^3^ Department of Health Services, Policy and Practice, Brown University School of Public Health, Providence, RI, United States; [omar_galarraga@brown.edu](mailto:omar_galarraga@brown.edu) [ORCID ID: 0000-0002-9985-9266]

^§^ Corresponding Author

Omar Galárraga, PhD

Associate Professor, Department of Health Services, Policy and Practice

Brown University School of Public Health

121 South Main Street, Box G-S121-2

Providence, RI United States

Phone: +1 (401) 863 2331

Email: [omar_galarraga@brown.edu](mailto:omar_galarraga@brown.edu)

**Databases searched:** PubMed (National Center for Biotechnology Information, Bethesda, Maryland), Econ-lit, (American economic Association, Nashville, Tennessee), supplemented by an Internet search of Google Scholar and search of cited references in publications identified via aforementioned database

**Date search was performed:** April 17, 2024

**Dates for which search was performed:** January 1, 2000 to March 31, 2024

**Full search strategy for PubMed (2,556 articles):**

Filters applied: January 1, 2000 -March 31, 2024, Full text/free full text availability

| Search | Query | Records retrieved |
| --- | --- | --- |
| #1 | *((HIV) OR (AIDS) OR (PLHIV))* | 196,875 |
| #2 | *((Medication Adherence[MeSH Terms]) OR (Patient compliance[MeSH Terms]) OR (Sustained Virologic Response[MeSH Terms]) OR (Retention in Care[MeSH Terms]) OR (Linkage to Care) OR (link to care) OR (linkage to treatment) OR (link to treatment) OR (Viral Suppression))* | 108,335 |
| #3 | *((Randomized Controlled Trial) OR (Controlled Clinical Trial) OR Clinical Trial))* | 476,337 |
| #4 | *((Reimbursement, Incentive[MeSH Terms]) OR (Financial Support[MeSH Terms]) OR (Cash) OR (Voucher) OR (Microfinance) OR (Financial incentive) OR (conditional economic incentive))* | 33,643 |
| #5 | *((Differentiated Care) OR (DSD) OR (Home delivery) OR (Community support) OR (Community Art Groups) OR (CAG) OR (multi-month dispensing) OR (MMD) OR (differentiated service models) OR (differentiated service delivery) OR (motivational interviewing) OR (MI))* | 1,108,033 |
| #6 | *((cost effectiveness) OR (cost analysis))* | 156,777 |
| #9 | #1, #2, #3,# 4 | 87 |
| #12 | #1, #2 , #4, #5 | 91 |
| #13 | #1, #2, #3, #5 | 1,107 |
| #15 | #1, #3,# 4 | 316 |
| #17 | #1, #3,# 4, #5 | 105 |
| #18 | #1, #2,# 4 | 215 |
| #19 | #1, #2,# 6 | 635 |

Search results for #9, #12, #13, #15, #17, #18, and #19 were used for screening.

**Full Search Strategy for EconLit (658 articles):**

*"HIV" "adherence" "economic strengthening" "sub-saharan africa"*

Filters applied: January 2000 – March 2024

**Full Search Strategy for Google scholar (821 articles):**

*HIV adherence AND DSD AND (reimbursement OR incentives OR financial support OR cash OR voucher OR microfinance OR Financial incentive) AND cost analysis*

Filters applied: 2000 - 2024
