# Supplementary material for: Cost-effectiveness of differentiated care models that incorporate economic strengthening for HIV antiretroviral therapy adherence: a systematic review
Source: Cost Eff Resour Alloc. 2024 May 24;22:46. doi: 10.1186/s12962-024-00557-w (PMC11127452; doi:10.1186/s12962-024-00557-w)
Supplement: Supplementary file 2 — Additional file 2: Quality assessment of full text articles that were standard health economic evaluations [file 12962_2024_557_MOESM2_ESM.docx]

Additional File 2 for the manuscript:

**Full Title:** Cost-effectiveness of differentiated care models that incorporate economic strengthening for HIV antiretroviral therapy adherence: a systematic review

**Running Title:** Cost-effectiveness of differentiated HIV care

**Authors:** Annie Liang ^1^; Marta Wilson-Barthes^2^; Omar Galárraga^3,§^

^1^ Brown University School of Public Health, Providence, RI United States; [annie_liang@brown.edu](mailto:annie_liang@brown.edu)

^2^ Department of Epidemiology, Brown University School of Public Health, Providence, RI United States; [marta_wilson-barthes@brown.edu](mailto:marta_wilson-barthes@brown.edu) [ORCID ID: 0000-0002-9845-7142]

^3^ Department of Health Services, Policy and Practice, Brown University School of Public Health, Providence, RI, United States; [omar_galarraga@brown.edu](mailto:omar_galarraga@brown.edu) [ORCID ID: 0000-0002-9985-9266]

^§^ Corresponding Author

Omar Galárraga, PhD

Associate Professor, Department of Health Services, Policy and Practice

Brown University School of Public Health

121 South Main Street, Box G-S121-2

Providence, RI United States

Phone: +1 (401) 863 2331

Email: [omar_galarraga@brown.edu](mailto:omar_galarraga@brown.edu)

**Quality assessment of full text articles that were standard health economic evaluations**

| **First Author (Cost effectiveness studies):** | **Shahmanesh M, et al., (2021)** | **Sibanda EL, et al., (2021)** | **Barnett PG, et al., (2009)** | **Tozan Y, et al., (2021)** | **Stevens ER, et al., (2018)** | | **Ekwunife OI, et al., (2021)** | | **Resch S, et al., (2022)** | | **Sahu M, et al., (2023)** | **Masiano S, et al., (2023)** | | **Choo J, et al., (2024)** | | **Gandhi A, et al., (2023)** |  |
| --- | --- | --- | --- | --- | --- | --- | --- | --- | --- | --- | --- | --- | --- | --- | --- | --- | --- |
| 1. Was the study objective presented in a clear, specific, and measurable manner? (7) | ✓ | ✓ | ✓ | ✓ | ✓ | | ✓ | | ✓ | | ✓ | ✓ | | ✓ | | ✓ |  |
|  |  |  |  |  |  |  | |  | |  | |  |  | |  | | |
| 2. Were the perspective of the analysis (societal, third party payer etc) and reasons for its selection stated? (4) |  | ✓ | ✓ | ✓ | ✓ | | ✓ | |  | |  |  | |  | |  |  |
|  |  |  |  |  |  | |  | |  | |  |  | |  | |  |  |
| 3. Were variable estimates used in the analysis from the best available source (ie RCT- Best, Expert Opinion- Worst) (8) | ✓ | ✓ | ✓ | ✓ | ✓ | | ✓ | | ✓ | | ✓ | ✓ | | ✓ | | ✓ |  |
|  |  |  |  |  |  | |  | |  | |  |  | |  | |  |  |
| 4. If estimates came from a subgroup analysis, were the groups pre specified at the beginning of the study? (1) | ✓ | ✓ | ✓ | ✓ | ✓ | | ✓ | | ✓ | | ✓ | ✓ | | ✓ | | ✓ |  |
|  |  |  |  |  |  | |  | |  | |  |  | |  | |  |  |
| 5. Was uncertainty handled by 1) stat analysis to address random events; 2) sensitivity analysis to cover a range of assumptions? (9) |  |  |  | ✓ | ✓ | | ✓ | | ✓ | | ✓ | ✓ | | ✓ | | ✓ |  |
|  |  |  |  |  |  | |  | |  | |  |  | |  | |  |  |
| 6. Was the incremental analysis performed between alternatives for resources and costs? (6) | ✓ | ✓ | ✓ | ✓ | ✓ | | ✓ | | ✓ | | ✓ | ✓ | | ✓ | | ✓ |  |
|  |  |  |  |  |  | |  | |  | |  |  | |  | |  |  |
| 7. Was the methodology for data abstraction (including value health states and other benefits) stated? (5) | ✓ | ✓ | ✓ | ✓ | ✓ | | ✓ | | ✓ | | ✓ | ✓ | | ✓ | | ✓ |  |
|  |  |  |  |  |  | |  | |  | |  |  | |  | |  |  |
| 8. Did the analysis horizon allow time for all relevant and important outcomes? Were benefits and costs that went beyond 1 year discounted (3-5%) and justification given for the discount rate? (7) | ✓ |  | ✓ | ✓ | ✓ | | ✓ | | ✓ | | ✓ |  | |  | | ✓ |  |
|  |  |  |  |  |  | |  | |  | |  |  | |  | |  |  |
| 9. Was the measurement of costs appropriate and the methodology for the estimation of quantities and unit costs clearly described? (8) | ✓ | ✓ | ✓ | ✓ | ✓ | | ✓ | | ✓ | | ✓ | ✓ | | ✓ | | ✓ |  |
|  |  |  |  |  |  | |  | |  | |  |  | |  | |  |  |
| 10. Were the primary outcome measure(s) for the economic evaluation clearly stated and were the major short term, long term and negative outcomes included? (6) | ✓ | ✓ | ✓ | ✓ | ✓ | | ✓ | | ✓ | | ✓ | ✓ | | ✓ | | ✓ |  |
|  |  |  |  |  |  | |  | |  | |  |  | |  | |  |  |
| 11. Were the health outcomes measures/scales valid and reliable? If previously tested valid and reliable measures were not available, was justification given for the measures/scales used? (7) | ✓ | ✓ | ✓ | ✓ | ✓ | | ✓ | | ✓ | | ✓ |  | |  | | ✓ |  |
|  |  |  |  |  |  | |  | |  | |  |  | |  | |  |  |
| 12. Were the economic model (including structure), study methods and analysis, and the components of the numerator and denominator displayed in a clear transparent manner? (8) |  |  |  |  | ✓ | |  | |  | |  | ✓ | | ✓ | |  |  |
|  |  |  |  |  |  | |  | |  | |  |  | |  | |  |  |
| 13. Were the choice of economic model, main assumptions and limitations of the study stated and justified? (7) |  |  |  |  | ✓ | |  | | ✓ | | ✓ | ✓ | | ✓ | | ✓ |  |
|  |  |  |  |  |  | |  | |  | |  |  | |  | |  |  |
| 14. Did the authors explicitly discuss direction and magnitude of potential biases? (6) | ✓ | ✓ | ✓ | ✓ | ✓ | | ✓ | |  | |  |  | | ✓ | |  |  |
|  |  |  |  |  |  | |  | |  | |  |  | |  | |  |  |
| 15. Were the conclusions/recommendations of the study justified and based on the study results? (8) | ✓ | ✓ | ✓ | ✓ | ✓ | | ✓ | | ✓ | | ✓ | ✓ | | ✓ | | ✓ |  |
|  |  |  |  |  |  | |  | |  | |  |  | |  | |  |  |
| 16. Was there a statement disclosing the source of funding for the study? (3) | ✓ | ✓ | ✓ | ✓ | ✓ | | ✓ | | ✓ | | ✓ |  | | ✓ | | ✓ |  |
|  |  |  |  |  |  | |  | |  | |  |  | |  | |  |  |
| Chiou Grading Total (/100): | 72 | 69 | 76 | 85 | 100 | | 85 | | 82 | | 82 | 73 | | 82 | | 82 |  |
